# A randomized study of 2 risk assessment models for individualized breast cancer risk estimation

**DOI:** 10.1093/jnci/djaf067

**Published:** 2025-04-01

**Authors:** Adrià López-Fernández, Laura Duran-Lozano, Guillermo Villacampa, Mónica Pardo, Eduard Pérez, Esther Darder, Anna Vallmajó, Rosa Alfonso, Mara Cruellas, Ariadna Roqué, Mireia Cartró, Adriana Bareas, Estela Carrasco, Alejandra Rezqallah, Ana Raquel Jimenez-Macedo, Sara Torres-Esquius, Maite Torres, Consol Lopez, Martín Espinosa, Alex Teulé, Elisabet Munté, Noemi Tuset, Orland Diez, Lidia Feliubadaló, Conxi Lázaro, Gemma Llort, Tim Carver, Lorenzo Ficorella, Nasim Mavaddat, Anna Mercadé, Antonis C Antoniou, Joan Brunet, Teresa Ramon y Cajal, Judith Balmaña

**Affiliations:** Vall d’Hebron Institute of Oncology (VHIO), Vall d’Hebron Barcelona Hospital Campus, Barcelona, Spain; Medical Oncology Department, Vall d’Hebron Barcelona Hospital Campus, Barcelona, Spain; Vall d’Hebron Institute of Oncology (VHIO), Vall d’Hebron Barcelona Hospital Campus, Barcelona, Spain; Oncology Data Science (ODysSey) Group, VHIO, Barcelona, Spain; Vall d’Hebron Institute of Oncology (VHIO), Vall d’Hebron Barcelona Hospital Campus, Barcelona, Spain; Genetic Counseling Unit, Oncology Service, Hospital del Mar, Barcelona, Spain; Vall d’Hebron Institute of Oncology (VHIO), Vall d’Hebron Barcelona Hospital Campus, Barcelona, Spain; Medical Oncology Department, Vall d’Hebron Barcelona Hospital Campus, Barcelona, Spain; Hereditary Cancer Program, Catalan Institute of Oncology, Josep Trueta University Hospital, IDIBGI, Girona, Spain; Genetic Counseling Unit, Arnau de Vilanova University Hospital, Lleida, Spain; Medical Oncology Department, Hospital de la Santa Creu i Sant Pau, Barcelona, Spain; Vall d’Hebron Institute of Oncology (VHIO), Vall d’Hebron Barcelona Hospital Campus, Barcelona, Spain; Medical Oncology Department, Vall d’Hebron Barcelona Hospital Campus, Barcelona, Spain; Hereditary Cancer Program, Catalan Institute of Oncology, Josep Trueta University Hospital, IDIBGI, Girona, Spain; Hereditary Cancer Unit, Corporació Sanitaria Parc Taulí, Sabadell, Spain; Vall d’Hebron Institute of Oncology (VHIO), Vall d’Hebron Barcelona Hospital Campus, Barcelona, Spain; Vall d’Hebron Institute of Oncology (VHIO), Vall d’Hebron Barcelona Hospital Campus, Barcelona, Spain; Medical Oncology Department, Vall d’Hebron Barcelona Hospital Campus, Barcelona, Spain; Vall d’Hebron Institute of Oncology (VHIO), Vall d’Hebron Barcelona Hospital Campus, Barcelona, Spain; Medical Oncology Service, Consorci Sanitari de Terrassa, Terrassa, Spain; Vall d’Hebron Institute of Oncology (VHIO), Vall d’Hebron Barcelona Hospital Campus, Barcelona, Spain; Medical Oncology Department, Vall d’Hebron Barcelona Hospital Campus, Barcelona, Spain; Medical Oncology Department, Hospital de la Santa Creu i Sant Pau, Barcelona, Spain; Breast Surgical Unit, Vall d’Hebron Barcelona Hospital Campus, Barcelona, Spain; Hereditary Cancer Program, Catalan Institute of Oncology—ICO, ONCOBELL—IDIBELL, L’Hospitalet de Llobregat, Spain; Centro de Investigación Biomédica en Red de Cáncer (CIBERONC), Instituto de Salud Carlos III, Madrid, Spain; Hereditary Cancer Program, Catalan Institute of Oncology—ICO, ONCOBELL—IDIBELL, L’Hospitalet de Llobregat, Spain; Genetic Counseling Unit, Arnau de Vilanova University Hospital, Lleida, Spain; Area of Clinical and Molecular Genetics, Vall d’Hebron Barcelona Hospital Campus, Barcelona, Spain; Hereditary Cancer Program, Catalan Institute of Oncology—ICO, ONCOBELL—IDIBELL, L’Hospitalet de Llobregat, Spain; Centro de Investigación Biomédica en Red de Cáncer (CIBERONC), Instituto de Salud Carlos III, Madrid, Spain; Hereditary Cancer Program, Catalan Institute of Oncology—ICO, ONCOBELL—IDIBELL, L’Hospitalet de Llobregat, Spain; Centro de Investigación Biomédica en Red de Cáncer (CIBERONC), Instituto de Salud Carlos III, Madrid, Spain; Hereditary Cancer Unit, Corporació Sanitaria Parc Taulí, Sabadell, Spain; Medical Oncology Service, Consorci Sanitari de Terrassa, Terrassa, Spain; Centre for Cancer Genetic Epidemiology, Department of Public Health and Primary Care, University of Cambridge, Cambridge, United Kingdom; Centre for Cancer Genetic Epidemiology, Department of Public Health and Primary Care, University of Cambridge, Cambridge, United Kingdom; Centre for Cancer Genetic Epidemiology, Department of Public Health and Primary Care, University of Cambridge, Cambridge, United Kingdom; Servei Veterinari de Genètica Molecular (SVGM), Universitat Autonòma de Barcelona, Cerdanyola del Vallès, Spain; Centre for Cancer Genetic Epidemiology, Department of Public Health and Primary Care, University of Cambridge, Cambridge, United Kingdom; Hereditary Cancer Program, Catalan Institute of Oncology, Josep Trueta University Hospital, IDIBGI, Girona, Spain; Hereditary Cancer Program, Catalan Institute of Oncology—ICO, ONCOBELL—IDIBELL, L’Hospitalet de Llobregat, Spain; Medical Sciences Department School of Medicine, University of Girona, Girona, Spain; Medical Oncology Department, Hospital de la Santa Creu i Sant Pau, Barcelona, Spain; Medical Oncology Department, Hospital Clínic de Barcelona, Barcelona, Spain; Vall d’Hebron Institute of Oncology (VHIO), Vall d’Hebron Barcelona Hospital Campus, Barcelona, Spain; Medical Oncology Department, Vall d’Hebron Barcelona Hospital Campus, Barcelona, Spain

## Abstract

**Background:**

Estimating breast cancer risk involves quantifying genetic and non-genetic factors. This supports health interventions and risk communication to ensure adherence to screening recommendations. This study evaluated the change in risk estimation when incorporating breast density and polygenic risk score (PRS) into the baseline cancer risk assessment and compared the efficacy of 2 risk-assessment delivery models.

**Methods:**

This 2-step study included 663 healthy women with a family history of breast cancer in which no pathogenic variants were identified. First, breast density and PRS were added to the baseline risk assessment for all participants. A randomized intervention study compared 2 delivery models (in-person vs pre-recorded video) for risk assessment in women at moderate or average risk. All tests were 2-sided.

**Results:**

Breast density and PRS reclassified the risk group into 33% of the participants, with only 5% reclassified as high-risk. After disclosure of their estimated multifactorial risk, 65% of women aligned their risk perception with their estimated risk, compared to 47% at baseline (*P* < .05). No statistically significant differences were found in the primary endpoint cancer worry, mean = 10.2 (SD = 3.1) vs 10.1 (2.7), between delivery models. In-person delivery had slightly better psychological outcomes (excluding the primary outcome) and higher satisfaction, though few participants in the video group sought in-person clarification.

**Conclusions:**

Incorporating breast density and PRS into risk assessments led to substantial reclassification, with 1 in 5 women facing de-escalated surveillance. Personalized assessments improve objective perceptions alignment. A model using a pre-recorded video-based model matches in-person delivery for moderate and average-risk women and is scalable for population-level implementation.

## Introduction

Breast cancer (BC) is a disease with a multifactorial etiology and heterogeneous complex genetic components.^1^ Over the past few decades, individuals with suspected hereditary cancer risk due to their family history have been offered multiplex cancer genetic testing. However, in 80–90% of index cases tested according to their personal or family history criteria,^2-4^ the genetic test does not reveal a pathogenic variant. Therefore, their healthy relatives are categorized as moderate risk, ie, with an approximate relative risk of 2, and they are recommended BC screening surveillance according to this estimation. There is a pressing need for more accurate tools to assess BC risk and to guide surveillance and prevention by incorporating all known genetic and non-genetic risk factors. The multifactorial predictive model named Breast and Ovarian Analysis of Disease Incidence and Carrier Estimation Algorithm (BOADICEA) includes age, family history, lifestyle/hormonal risk factors,^2-6^ monogenic germline pathogenic variants,^7^^,^^8^ common low-risk variants,^9-14^ body mass index,^15^^,^^16^ alcohol intake,^17^ and breast density^16^ in the CanRisk tool.^18-20^ Integration of the polygenic risk score (PRS) into the model has recently been shown to improve its predictive performance.^21^^,^^22^

Before implementing this multifactorial and complex personalized risk estimation in the context of cancer genetics clinics, we novelly aimed to investigate the impact of adding both the mammographic breast density and PRS to the baseline CanRisk-based BC risk estimation (ie, based on family history, lifestyle, and hormonal factors). We hypothesized that a small change in risk stratification would not justify adding such a level of complexity to risk estimation and subsequent risk communication. In fact, some women may have reservations about personalized BC risk assessment for a variety of reasons, such as differing perspectives on the quantified value of genetic and non-genetic factors,^23^ misunderstanding of the risk, a mismatch between risk perception and quantified objective estimation,^24^ or other personal considerations. Furthermore, communicating risk is inherently challenging due to the uncertainty involved, potential variability in professional training, the complexity involving statistical information, individuals’ numeracy, or other reasons.^25^ Finally, providing structured, homogenous information to all women ensures consistency, simplifies the communication process, enhances accessibility, and reduces confusion. However, it may lack personalization, reduce engagement, oversimplify complex issues, and face resistance from those preferring personalized guidance. However, in-person delivery of BC risk estimation and surveillance recommendations is unlikely to be affordable at the population level. This highlights the need to evaluate the effectiveness of alternative non-in-person delivery methods. We hypothesized that there would be no differences in psychological outcomes when delivering the results in-person vs with a pre-recorded video in women who are in the average or moderate BC risk group.

The PRiSma study prospectively evaluated the uptake and impact of tailored BC risk stratification in healthy women with a family history of BC. In the first phase, the study aimed to (1) evaluate how the addition of breast density and PRS to non-genetic risk factors changes risk stratification in risk assessment and (2) determine the change in alignment between self-perceived BC risk and objective quantification after undergoing cancer risk assessment. In the second phase, a randomized study was conducted to (3) assess and compare the psychological impact and satisfaction of the 2 cancer risk assessment delivery models.

## Methods

The PRiSma study was a multicenter research project conducted in Spain involving unaffected women who visited 7 cancer genetics clinics of the Catalan Oncology Network.

### Study design

Participants were consecutively recruited from cancer genetics clinics where their initial risk assessment (baseline) was conducted based on lifestyle/hormonal risk factors and family history using the CanRisk tool^18^ between August 2020 and December 2021. Informed consent was provided in person during the first visit for the study, where demographics, hormonal, and other risk factor data were collected. If a mammogram was not available at this time, each participant underwent one breast density determination by mammogram using the BI-RADS classification. In addition, a blood sample was collected for genotyping single nucleotide polymorphisms (SNPs), enabling the calculation of breast cancer PRS. Individual risks were re-estimated by incorporating at least one additional risk factor: breast density and/or PRS (exposure). Participants were then categorized into average, moderate, or high-risk groups based on their 10-year BC risk according to the NICE thresholds^26^ (<3%, 3%-8% and >8%, respectively). In the second phase of the study, individuals with average or moderate risk were randomized into 2 risk delivery models: in-person (control arm) or through a pre-recorded video (experimental arm), with the option to request a sequential in-person visit. Delivery of BC risk estimation, early detection recommendations, and prevention options were performed in person among those classified as high risk. Participants were asked to complete questionnaires to assess cancer worry, risk perception, numeracy skills, personality traits, satisfaction, knowledge, and the psychological impact of undergoing a multifactorial BC risk assessment.

### Population

Eligible participants were women between 30 and 65 years of age who had not been diagnosed with BC, were not carriers of a pathogenic variant in BC susceptibility genes, and had at least one first-degree relative diagnosed with BC. We included (1) unaffected women who tested negative on the genetic test performed on themselves (either BC panel testing or for a known familial pathogenic variant in *ATM* or *CHEK2*) who were offered participation in person; and (2) first-degree relatives of the affected women, who tested negative on the BC panel, and who were contacted by telephone and invited to participate, followed by an in-person visit. Multigene BC panel testing was performed according to national clinical criteria.^27^ Panel testing was deemed negative when the test did not identify any pathogenic variants or when the test identified a variant of uncertain significance.

### Genotyping

Genotyping determined PRiSma PRS_268_, which included 268 SNPs from the validated BC PRS_313_ (PGS-ID: PGS000004), and both have previously been described^21^ (Supplementary Methods, Tables S1 and S5). The CanRisk tool was modified to allow for PRiSma PRS268.

All participants received risk assessment and surveillance recommendations from certified professionals. All the participants signed an informed consent form. The study was approved by the Vall d’Hebron Hospital Ethics Committee (PR(AG)516/2019) and the ethics committees of each institution.

### Breast cancer risk estimation

For this study, the factors included in the CanRisk tool were weight, height, menarche, menopause age (if applicable), number of children, age at first child, breast density (in women older than 35 years), cancer family history, negative result of panel testing for the major BC genes, and BC-associated PRS_268_.

### Data collection and outcomes

Demographic data, family history, and genetic data were sourced from the electronic medical records of each recruiting center. The outcomes were defined as the change in BC risk group classification after including breast density and PRS to non-genetic risk factors in the risk assessment, the change in the alignment between subjective risk perception and objective risk estimation, and the scores on psychological scales.

We measured the alignment of subjective risk perception with objective risk estimation by comparing individuals' risk perception with their estimated quantified risk group category (categorized as average, moderate, or high risk) at baseline and after undergoing cancer risk assessment, regardless of the delivery model. Participants categorized their own risk perception into 1 of the 3 groups: average, moderate, or high risk.

This study employed various psychological assessments to evaluate multiple dimensions. At the time of enrollment in the study, cancer worry was assessed using the Cancer Worry Scale (CWS),^28^ numeracy skills using the Lipkus Numeracy Scale,^29^ and personality traits using the Mini-IPIP tool.^30-32^ Risk perception was also assessed as detailed above. After the personalized risk assessment, the psychological impact of personalized BC risk estimation was evaluated using an adapted Multidimensional Impact of Cancer Risk Assessment Questionnaire (MICRA, including distress, uncertainty, and positive experiences).^33^ Decisional conflict was assessed using the Decisional Conflict Scale,^34^^,^^35^ and anxiety levels were assessed using the State-Trait Anxiety Inventory.^36-38^ Risk perception was reassessed at this time point. Additionally, custom-designed questions with 5-point Likert scale responses explored satisfaction with disclosure and understanding of BC risk causes. Pre- and post-intervention questionnaires were administered using RedCap^39^^,^^40^ (more information in Supplementary Methods).

### Randomization of 2 delivery models for results disclosure

After the risk-group categorization, participants in the average and moderate risk categories were randomized (1:1) into 2 delivery models for results disclosure, one in-person (control arm) and one based on a risk-adjusted pre-recorded video (intervention). Participants were informed after the initial in-person visit that they would be randomized to receive the results either in person or through a pre-recorded video sent by email. The randomization was stratified by the center. Two pre-recorded videos were created, each tailored to a specific risk group. All patients with a high-risk estimate received their results in person.

### Video development and content

The videos were created through a participative process involving genetic healthcare professionals and health and communication experts. The content was structured into 3 parts: (1) a brief introduction to the PRiSma study, (2) an overview of the multifactorial causes of breast cancer, and (3) a personalized risk assessment, which was tailored to the participant's risk group. In this third section, participants’ risk was explained in terms of both short-term and long-term projections, with comparisons to the population average. The content varied for individuals at average or moderate risk, providing specific recommendations for surveillance and prevention based on their risk level. Each video was approximately 4 minutes long, designed to convey complex information in an accessible and engaging manner using visual aids like infographics.

### Statistical analysis

A descriptive analysis was used to summarize the individuals’ characteristics. The study was designed without a formal sample size calculation. Instead, it aimed to recruit all eligible patients over an 18-month period. At the time of the study’s conception, no robust prior information was available to define a hypothesis testing. Consequently, no formal statistical power was predefined. The primary endpoint for the non-randomized part of the study was the percentage of change on risk group classification after incorporating breast density and PRS. For the randomized part, the primary endpoint was to estimate the difference in the mean change in CWS values from baseline to post-disclosure between the 2 groups. The secondary endpoints were the scores in the MICRA scale, the State-Trait Anxiety Inventory, the Decisional Conflict Scale, and the satisfaction with the delivery method. To account for the non-normal distribution of values, the non-parametric Wilcoxon test was used to compare the MICRA, State-Trait, and CWS scores between the 2 randomized groups. To study the association between baseline factors and psychological impact, a multivariate analysis was conducted using MICRA total scores as the outcome. A generalized linear effect model was used for this analysis. We used a negative binomial distribution (natural-log link function) to account for the non-normal, right-skewed distribution of MICRA values. The McNemar test was used to evaluate the association between adequacy of cancer risk perception before and after the intervention. Fisher’s exact test was used to statistically compare whether the distribution of risk groups changed after incorporating breast density and PRS. Data imputation was not performed. All tests were 2-sided with a value of *P* < .05 considered statistically significant without adjustment for multiple comparisons due to the exploratory nature of the study. All statistical analyses were performed using the R statistical software.

## Results

**Figure 1.**
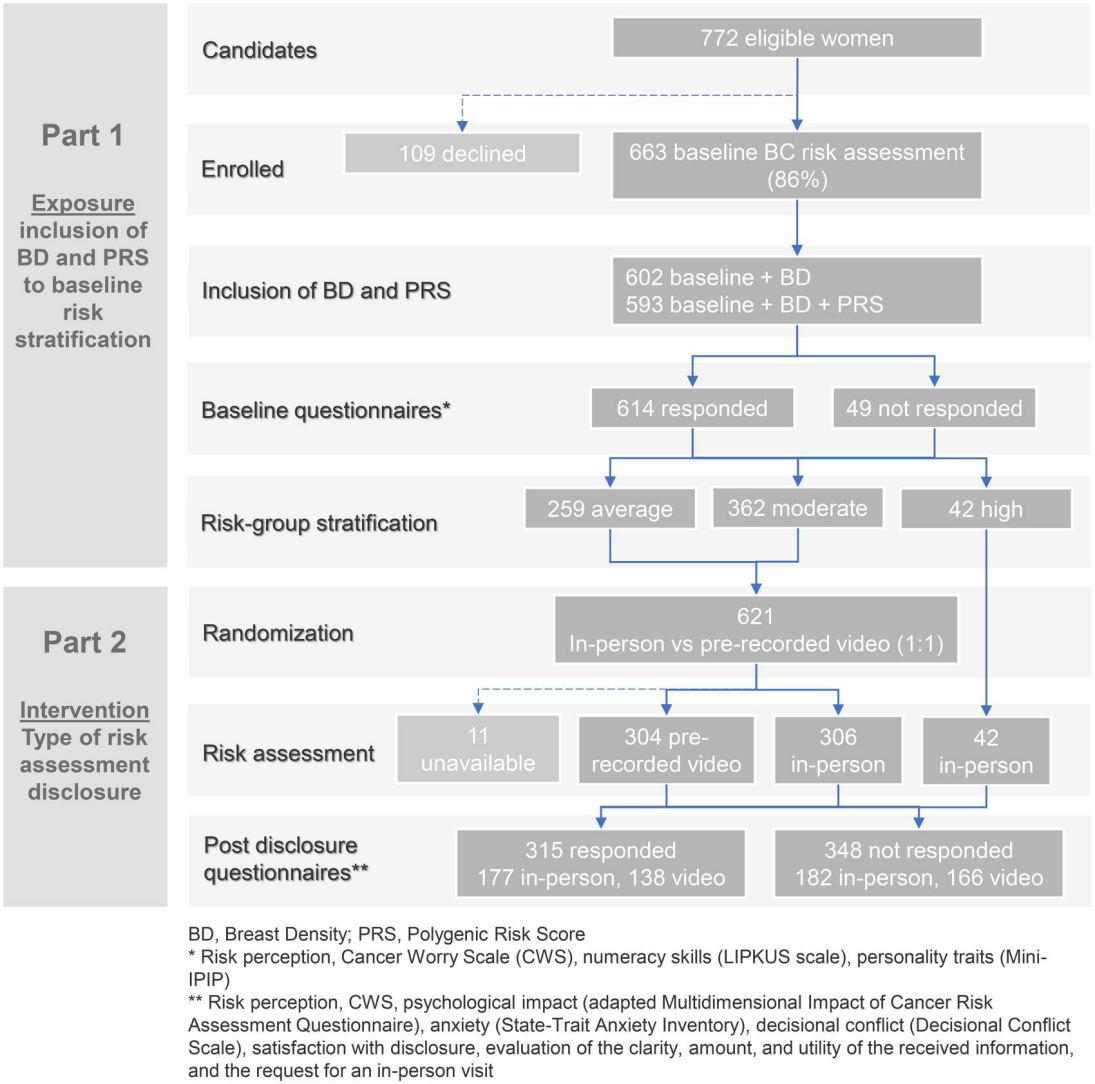
CONSORT flow diagram of participant stratification by risk group and subsequent randomization for delivery of risk assessment.

Out of 772 eligible individuals contacted, 663 (86%) agreed to participate. Breast density was assessed in 91% of the participants, and PRS was calculated for 98%. Overall, we obtained data for 593 participants, including non-genetic risk factors, breast density, and PRS. For the questionnaires, 92% of the participants completed the first phase and 47% completed the second phase (Figure 1). Briefly, 97% of the participants were of European ancestry, the median age was 45 years, and 70% were premenopausal. Breast density was mostly distributed between categories B and C (Table 1).

**Table 1.** Characteristics of the study population, including overall population, randomized groups (average-risk and moderate-risk), and non-randomized group (high-risk).

		Moderate and average risk (*n* = 621)		High risk (*n* = 42)
	All participants	Risk assessment in-person	Risk assessment with pre-recorded video	Risk assessment in-person
	*n* (%)	*n* (%)	*n* (%)	*n* (%)
Total	663	306	304	42
Age^a^	46 (8.4)	46.2 (8.5)	46.4 (8.3)	45.6 (6.2)
Ancestry				
European	645 (97)	299 (97)	298 (98)	39 (93)
Asian	1 (0.1)	0 (0)	1 (0.33)	0 (0)
Hispanic/Latina	15 (2)	6 (2)	5 (2)	3 (7)
Black/African	0 (0)	0 (0)	0 (0)	0 (0)
Ashkenazi Jewish	1 (0.1)	0 (0.5)	0 (0)	0 (0)
Others/admixed	1 (0.1)	1 (0.5)	0 (0)	0 (0)
Age of menarche	12.7 (1)	12.8 (1)	12.7 (1)	12.4 (1)
Menopause status				
Premenopausal	461 (70)	210 (69)	209 (69)	33 (79)
Menopausal	202 (30)	96 (31)	95 (31)	9 (21)
Parity				
No children	153 (23)	70 (22)	72 (24)	11 (26)
1 child	172 (26)	69 (23)	91 (30)	8 (19)
2 children	289 (44)	143 (47)	119 (39)	20 (48)
More than 2 children	49 (7)	24 (8)	22 (7)	3 (7)
Body mass index				
<18.5	24 (4)	13 (4)	7 (2)	5 (12)
18.5–24.9	385 (58)	175 (57)	175 (58)	26 (62)
25–29.9	171 (26)	72 (23)	87 (29)	11 (26)
>29.9	83 (13)	46 (14)	35 (12)	0 (0)
Breast density				
A category	26 (4)	14 (5)	12 (4)	0 (0)
B category	247 (37)	119 (39)	124 (41)	2 (5)
C category	241 (36)	120 (39)	102 (33)	13 (31)
D category	86 (13)	25 (8)	36 (12)	27 (64)
Non-available	63 (10)	28 (9)	30 (10)	0 (0)
10-year baseline cancer risk estimation				
Average (<3%)	258 (39)	137 (45	117 (39)	–
Moderate (3-8%)	360 (55)	169 (55)	187 (61)	–
High (>8%)	42 (6)	–	–	42 (100)
Numeracy skills^a^^,^^b^	7 (2.1)	7 (2.1)	7 (2.2)	7 (2.3)
Personality traits^a^^,^^c^				
Neuroticism	8 (3)	8 (3)	8 (3)	8 (2)
Conscientiousness	11 (3)	12 (3)	12 (3)	12 (3)
Extraversion	9 (3)	9 (3)	9 (3)	8 (3)
Agreeableness	12 (2)	12 (3)	13 (2)	13 (2)
Openness	10 (3)	10 (3)	10 (3)	11 (2)

a Mean and standard deviation.

b Lipkus scale: scores between 0 and 9.

c Personality traits: each factor scores between 0 and 16.

### Change of risk group with the addition of breast density and PRS

**Figure 2.**
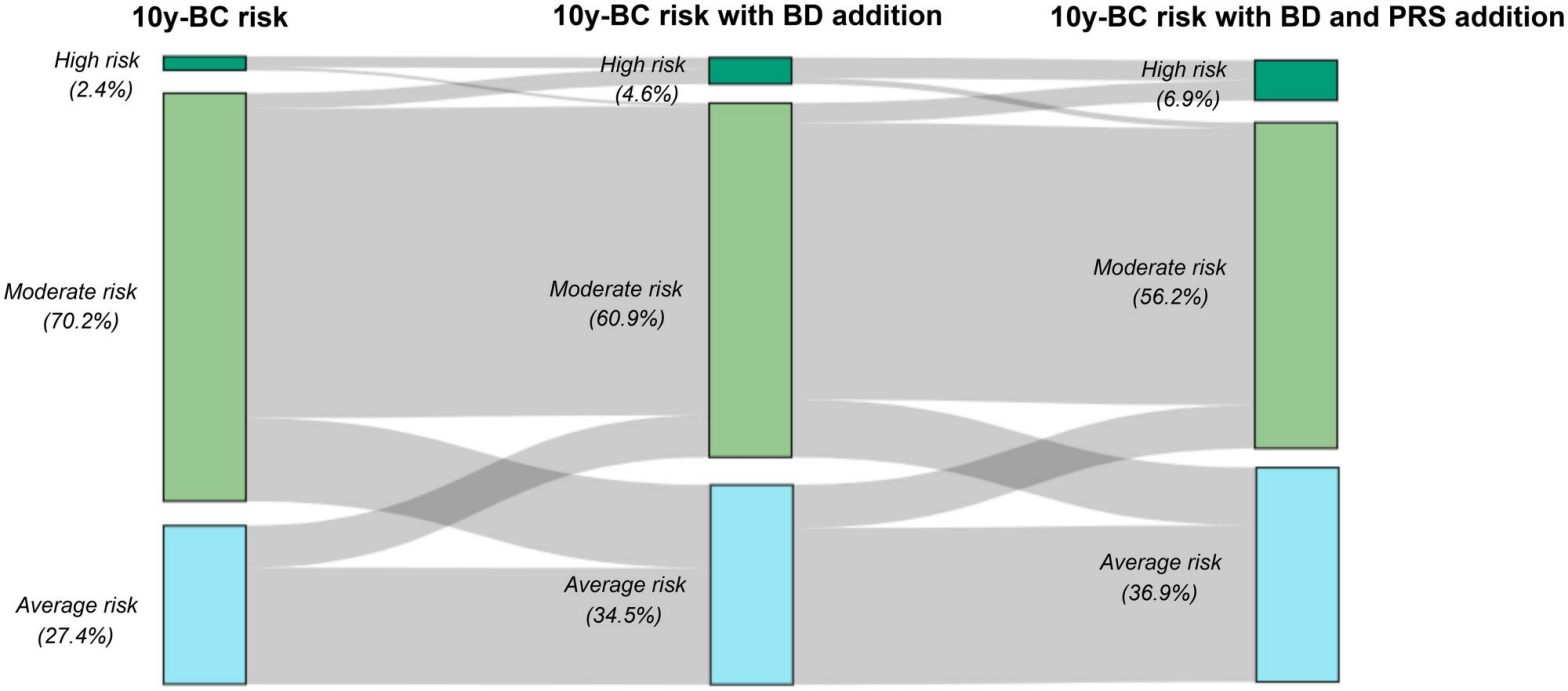
Change in 10-y BC risk stratification after addition of breast density and PRS to non-genetic risk factors of the 395 participants with all data available. BC = breast cancer; BD = breast density; PRS = Polygenic Risk Score.

With the initial baseline 10-year risk estimation, 2.5% of individuals were classified as high risk, 70% as moderate risk, and 27.5% as average risk. After including breast density in the risk estimation, the percentages were 4.5%, 61%, and 34.5%, respectively. Finally, when incorporating breast density and PRS information into the full model, the percentage of patients classified as high, moderate, and average risk was 7%, 56%, and 37%, respectively (Fisher’s exact test, *P* < .001, compared with the baseline assessment) (Figure 2).

Overall, 198 of 593 individuals (33%) experienced a change in their 10-year risk category when adding breast density and PRS to the baseline estimation. Specifically, 19% had a reduction in their 10-year risk category, whereas 14% experienced an increase, with only 5% being reclassified as high-risk category (Tables S2 and S3).

### Alignment between risk perception and risk estimation

**Figure 3.**
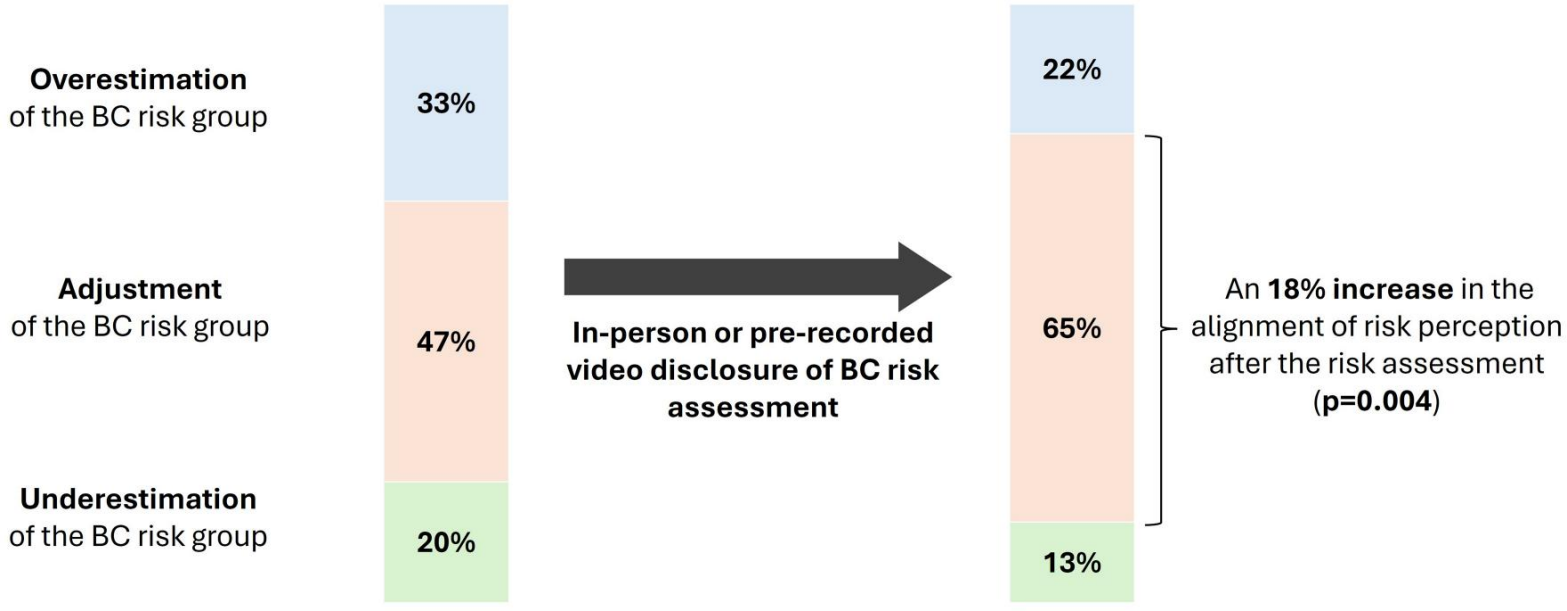
Rate of adequacy between subjective BC risk perception and objective BC risk estimation before and after individualized BC risk assessment visit.

At baseline, 47% of the participants exhibited a concordance between their perceived risk and the objective estimated group risk, while 33% overestimated their risk of developing BC. Following the personalized risk assessment intervention, the proportion of participants with an aligned risk perception increased to 65%, indicating an 18% improvement in concordance between cancer risk perception and objective estimation (*P* < .05) (Figure 3). The improvements in concordance did not differ between the delivery models of risk assessment.

### Psychological impact of the personalized breast cancer risk estimation

The high-risk group had statistically significantly higher MICRA total scores compared to the moderate and average groups (29.0 vs 16.7 and 13.4, respectively, *P* < .001), indicating greater negative reactions. Distress was also higher in the high-risk group than in the moderate- and average-risk groups (9.3 vs 3.7 and 3.0, respectively, *P* < .001). No statistically significant differences were found in uncertainty scores. Positive experiences were lower in the high-risk group than in the moderate and average groups (8.4 vs 11.7 and 14.9, respectively; *P* < .001). Cancer worry was higher in the high-risk group than in the other 2 groups (12.2 vs 10.1 and 10.3, respectively, *P* < .001). Participants reported a very low score in the decisional conflict scale (mean 8; SD 9.3) and no regret for having undergone personalized breast cancer risk assessment (mean 0.1; SD 0.6). Across the risk groups, there were no statistically significant differences in state anxiety, decisional conflict, regret, understanding of BC etiology, perception of the risk-estimation benefit, or willingness to recommend others to perform a BC risk-estimation intervention (Table 2).

**Table 2.** Psychological outcomes after delivery of BC risk estimation according to delivery model and risk group.

	Delivery model			Risk group			
Psychological outcome (range)	In-person (*n* = 306) Mean (SD)	Pre-recorded video (*n* = 304) Mean (SD)	*P* value^a^	Average risk (*n* = 258) Mean (SD)	Moderate risk (*n* = 360) Mean (SD)	High risk (*n* = 42) Mean (SD)	*P* value^a^
MICRA total (0-105)	13.2 (11.6)	17.7 (12.5)	.001	13.4 (12.2)	16.7 (12.1)	29 (12.3)	<.001
MICRA distress (0-30)	2.9 (3.9)	4 (4.3)	.02	3 (3.9)	3.7 (4.3)	9.3 (7.1)	<.001
MICRA uncertainty (0-45)	5.1 (6.1)	6.7 (7.6)	.03	6 (6.8)	5.7 (6.9)	8.8 (6.7)	NSS
MICRA positive experiences (0-20)	13.8 (5.1)	12.1 (5.7)	.005	14.9 (5.1)	11.7 (5.3)	8.4 (2.4)	<.001
MICRA regret (0-5)	0.04 (0.2)	0.2	.04	0.09 (0.6)	0.1 (0.6)	0.04 (0.2)	NSS
Cancer Worry Scale (6-24)	10.2 (3.1)	10.1 (2.7)	NSS	10.3 (3)	10.1 (2.8)	12.2 (3.6)	<.01
State Anxiety Scale (0-60)	15.5 (10.2)	16.7 (10.4)	NSS	15.8 (10.8)	16.2 (9.9)	18.3 (7)	NSS
Decisional conflict Scale (0-64)	8.8 (10.5)	7.1 (6.8)	NSS	7.3 (8.7)	8.4 (9.1)	7.7 (13.1)	NSS
Understanding that BC risk has a multifactorial etiology (0-5)^b^	4.67 (0.75)	4.58 (0.8)	NSS	4.5 (0.8)	4.7 (0.8)	5 (0)	<.05
Perceived benefit of undergoing BC risk estimation (0-5)^b^	4.2 (1)	4.1 (1)	NSS	4.2 (0.9)	4.1 (1.1)	4.2 (0.9)	NSS
Recommend others to undergo BC risk estimation (0-5)^b^	4.7 (0.7)	4.5 (0.8)	NSS	4.6 (0.8)	4.5 (0.8)	4.6 (0.7)	NSS

MICRA = Multidimensional Impact of Cancer Risk Assessment; NSS = Not statistically significant.

a Kruskal–Wallis test.

b 5-point Likert scale.

### Comparison between the 2 delivery models

Participants categorized into the average- or moderate-risk groups were randomly assigned to have their results disclosed in-person or through a pre-recorded video (*n* = 621). Their characteristics and psychological outcomes are detailed in Tables 1 and 2, respectively.

**Figure 4.**
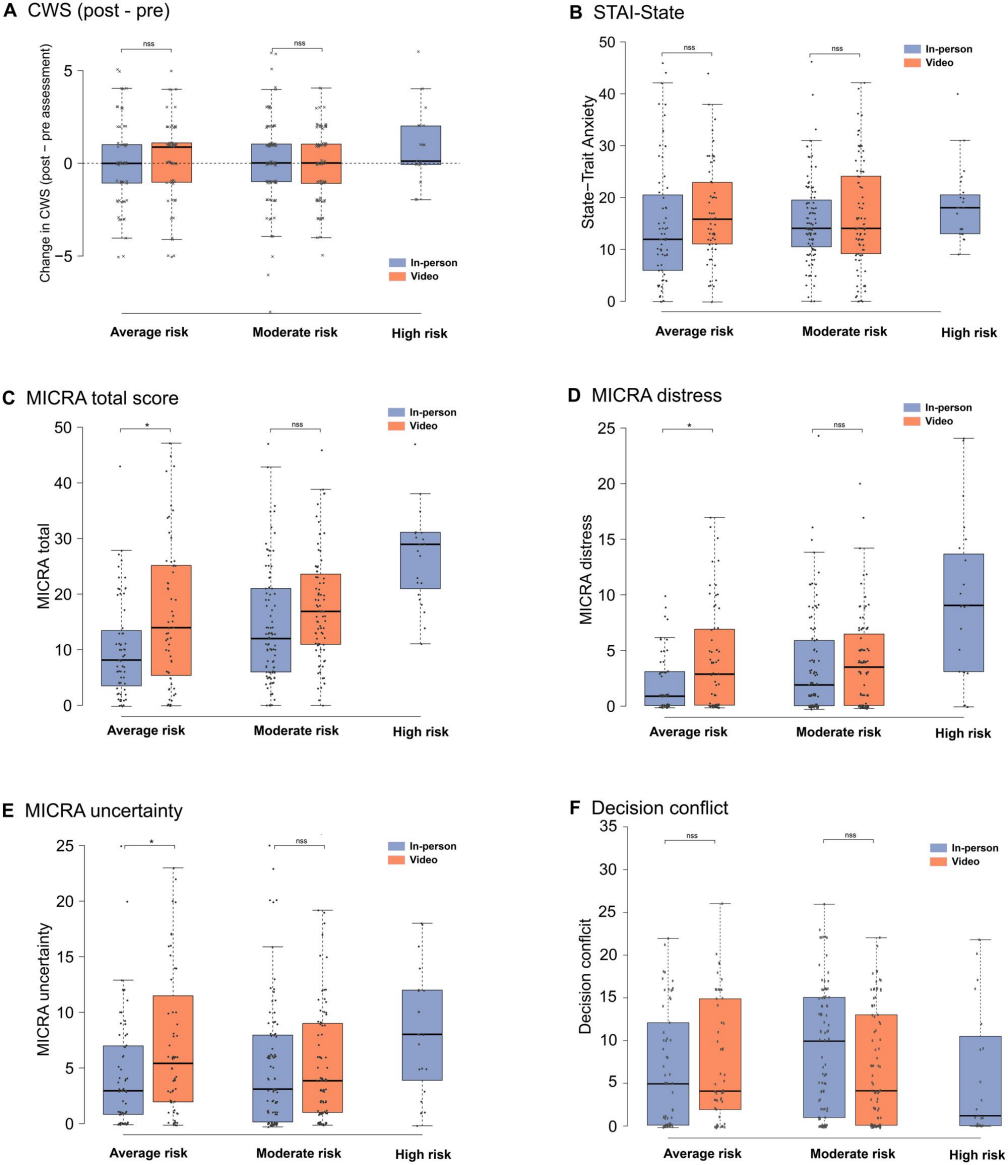
Psychological scores after BC risk assessment according to risk category and delivery model. (A) Change in Cancer Worry Scale. (B) State-Trait Anxiety scale: state scores are represented. (C) MICRA total scores. (D) MICRA distress. (E) MICRA uncertainty. (F) Decisional conflict. CWS = Cancer Worry Scale; MICRA = Multidimensional Impact of Cancer Risk Assessment Questionnaire.

We aimed to analyze whether cancer worry differed according to the cancer risk category and whether any of the risk assessment delivery models affected any change in cancer worry compared to the baseline score. To do so, we assessed the differences between the post-disclosure and baseline CWS in each group. We did not find differences in CWS between women categorized as having a moderate or average BC risk. Similarly, there were no statistically significant differences in CWS between the 2 delivery methods. For anxiety scores, no differences in anxiety state were observed across study participants (Figure 4).

For the MICRA scores, individuals in the pre-recorded video group exhibited slightly higher scores than those in the in-person group (median 16.0 vs 10.0), with statistical significance only among individuals in the average-risk group (*P* < .05). This trend was consistent with that of the distress and uncertainty subscales. Interestingly, only 1% of participants who received their results via the pre-recorded video requested a further in-person visit for clarification.

The multivariate analysis showed that receiving results via pre-recorded video, being categorized into a high-risk group, and having lower levels of numeracy skills were associated with higher MICRA scores, indicating a greater psychological impact (*P* < .05). Personality traits were not linked to increased psychological impact after the risk assessment (Table S4).

**Figure 5.**
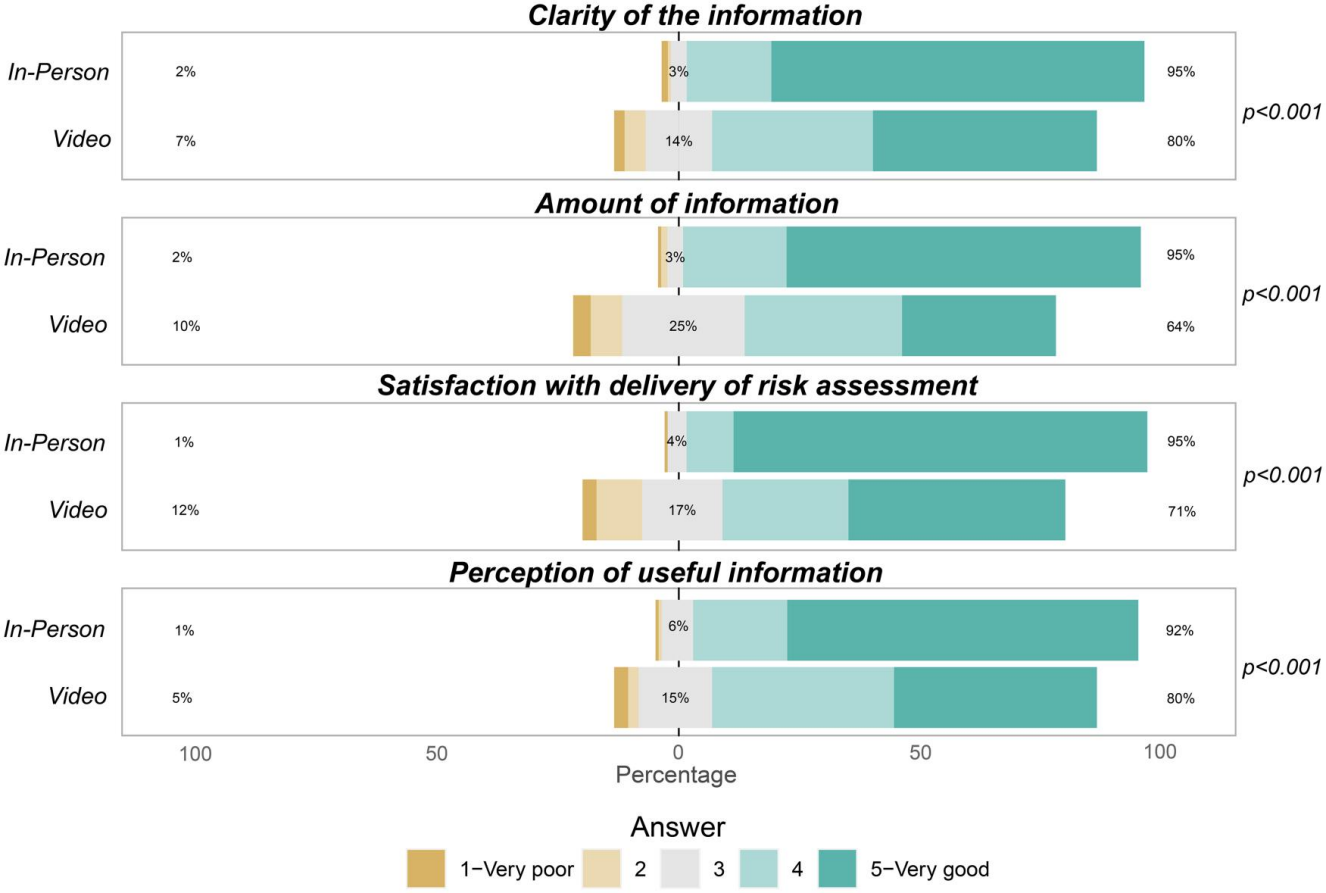
Comparison of delivery models in terms of quality of information and satisfaction with delivery model (average and moderate risk).

Participants who received their risk assessment via the pre-recorded video indicated fewer “good” or “very good” scores in clarity of information (80% vs 95%, *P* < .001), amount of information received (64% vs 95%, *P* < .001), and perceived utility of the information (80% vs 92%, *P* < .001) compared to participants in the in-person group. Satisfaction with disclosure via pre-recorded video was also lower compared to the in-person model (71% vs 95%, *P* < .001) (Figure 5).

## Discussion

Our study evaluated the clinical impact of comprehensive BC risk assessment within a cohort of healthy women with a family history of BC and negative germline genetic results. Furthermore, we compared the psychological outcomes and satisfaction levels associated with the 2 different risk assessment delivery models.

The inclusion of breast density and PRS in the BC risk estimation led to changes in risk categories in 33% of women, with 14% experiencing an increase in their risk category and 19% a decrease. After the risk assessment intervention, 65% of the participants demonstrated an alignment between their risk perception and objective risk estimation, reflecting an 18% improvement compared to the baseline. Women experiencing a numerically higher psychological impact were those with elevated predicted risk, those whose risk assessment was delivered via a pre-recorded video, and those with lower numeracy skills. Notably, the delivery of results in person was associated with slightly better psychological outcomes compared to a pre-recorded video, although no statistically significant differences were detected in reported levels of anxiety or decisional conflict. Furthermore, very few participants requested an in-person visit for clarification. The addition of PRS to improve the accuracy of BC risk estimation is relatively recent, and few studies have assessed the uptake of PRS-based BC risk assessment, with acceptance ranging between 62% and 88%.^41-43^ Our higher uptake of 86% might be a consequence of a highly motivated population to decipher their BC risk due to their family history. In our study, the risk assessment intervention was performed by professionals well-versed in the needs of healthy women with a family history of cancer, which might have compensated for the potential pitfalls of multifactorial risk-based assessments. The change in risk classification using the full BOADICEA model in our study population was comparable to the changes observed in other studies.^44-47^ This reclassification is mostly observed among women initially classified as moderate risk due to their family history and could help de-escalate screening for those shown to be at a lower risk while increasing surveillance for those who need it the most. In our analysis, we observed that the addition of PRS would de-escalate surveillance recommendations for almost 1 out of 5 patients, which might contribute to a more cost-effective strategy for the early detection and prevention of BC.^48^

Risk assessment interventions have been shown to align patients’ risk perceptions with their objective levels of risk.^49^ At baseline, more than half of the women over- or underestimated their risk of developing BC. After personalized risk assessment, for 65% of women, there was a close correlation between risk perception and risk categorization. Investing time in disclosing BC risk improves the accuracy of self-risk perception^50^ and is a key factor in promoting adherence to surveillance.^51^^,^^52^ In line with others, we emphasize that communication strategies should be meticulously crafted, particularly within the de-escalation subgroup, where evidence-based recommendations are essential for gaining acceptance among women.^53^

Our findings suggest that the overall psychological impact following comprehensive BC risk estimation appears to be low, which is consistent with prior experiences.^54^ Women classified as high-risk exhibited some psychological impact with scores, such as individuals carrying a high-risk pathogenic variant^55-57^ and an increase in cancer worry. This suggests that high-risk designation may influence cancer-related anxiety, and this group might benefit from in-person disclosure of cancer risk assessments.

One of the main challenges in implementing risk-based BC screening at the population level is the notable resources required to provide individualized risk assessments and communicate the results effectively, ensuring that women fully understand their risks and can engage in shared decision-making about the type of screening. Our study compared the psychological impact, understanding, and satisfaction of in-person vs pre-recorded videos for the delivery of risk assessment. We did not identify statistically significant differences in anxiety or cancer worry between the 2 delivery methods even though in-person disclosure was scored as more satisfactory with more quality information than the pre-recorded video. Overall, these findings suggest that, among women at moderate or average risk, both delivery methods can effectively communicate results without substantially increasing psychological distress. Indeed, only 1% of the participants in the pre-recorded group requested a subsequent in-person visit.

This study had some limitations. First, PRS_313_ (from which PRS_268_ derives) was developed and validated in women of European ancestry,^58^^,^^59^ and its performance cannot be applied in a multi-ethnic population.^60^ Second, our high uptake for individualized BC risk assessment might have been enriched due to a population highly motivated by their family history, which does not guarantee the same level of acceptance in the general population. Finally, the study does not allow us to determine whether the lower satisfaction with the video intervention was due to the absence of in-person interaction or was inherent to the video content itself. Further research is needed to explore this area in depth. A key strength of the study was the randomization of the intervention between the 2 delivery models, which provides evidence for the promotion of scaling up at the population level.

## Conclusions

Our results underscore the high acceptance of personalized BC risk estimation among women with a family history of BC. This conclusion was supported by 3 key indicators: a high rate of participation, low levels of decisional conflict post-participation, and a notable absence of regret among participants. The addition of breast density and PRS analysis led to substantial reclassification of the baseline cancer risk. In addition, the overall psychological impact was low. Delivery of results with pre-recorded videos among women at average and moderate risk revealed no differences in psychological outcomes compared with in-person visits. Overall, this study provides evidence that supports the feasibility of implementing personalized BC risk stratification and screening.

## Supplementary Material

djaf067_Supplementary_Data

## Data Availability

For this research, the data will be shared upon request. The data available for sharing included genotyping information, carrier status, breast density, hormonal exposure, and psychological outcomes. Any shared data will be codified to ensure the anonymity of the participants. The data will be made available under conditions that ensure confidentiality and privacy of the participants, with no direct identifiers included. The authors will consider requests for data sharing and analyses will be permitted after a request is approved. To request access to data, please contact Dr Judith Balmaña at jbalmana@vhio.net. There are no restrictions on the types of analyses permitted once a request is approved. If data sharing is not possible, the authors will provide a reason for refusal.
