# Supplementary material for: A randomized study of 2 risk assessment models for individualized breast cancer risk estimation
Source: J Natl Cancer Inst. 2025 Apr 1;117(8):1593–604. doi: 10.1093/jnci/djaf067 (PMC12342814; doi:10.1093/jnci/djaf067)
Supplement: djaf067_Supplementary_Data [file djaf067_supplementary_data.zip › djaf067_Supplementary_Data/Supplementary methods.docx]

**Supplementary methods**

1. PRS Genotyping and calculation

PRiSma PRS contains a total of 268 SNPs; 231 from the 313 SNP PRS (Mavaddat et al., 2019) and 37 surrogates in high linkage disequilibrium (r^2^>0.9) with variants from PRS 313. The remaining 55 SNPs could not be genotyped with Taqman probes. Custom probes were designed for 29 SNPs (Supplementary table 5). The proportion of the polygenic variance explained by the PRS, estimated a using a retrospective likelihood approach (α_RL_) of PRISMA PRS_268_ is 0.396 and BCAC PRS_313_ is 0.441^1^. Genotyping was performed using two TaqMan OpenArray plates (ThermoFisher). Amplification was performed using the QuantStudio 12K Flex Real Time PCR System (ThermoFisher). As for quality control call rate per sample and per SNP were calculated, and an 80% threshold was established to discard samples with lower calling. For 263/268 SNPs (98% variants) the observed call rate was above 90%, lowest call rate being 84%. Ninety-six percent of the samples (651/678) had a SNPs amplification call rate over 90%. A total of 18 samples had a call rate between 80% and 90%, and 9 samples were discarded for presenting a call rate under 80%.

VCF files containing allele dosage were generated and uploaded to the online tool CanRisk, which was extended to include the PRISMA_268_ as an optional, alternative PRS in the BOADICEA v6 algorithm. In the case of missing genotyping information (e.g., due to non-amplification or cluster overlapping), genotype was imputed according to 2*q*(1-q)+2*q^2 considering the Hardy-Weinberg principle and the observed effect allele frequency in BCAC studies (q). PRS was calculated following the log-additive model (β_1_x_1_+β_2_x_2_+…+β_k_x_k_+β_n_x_n_) where β_k_ is the per-allele log odds ratio (OR) for breast cancer associated with SNP k, x_k_ is the risk allele dosage for SNP k, and n is the total number of SNPs included in the PRS (268 in this case). The effect allele dosage (x_k_) was obtained from the SNPs genotyping, ranging from 0 to 2. More details can be found in Mavaddat et al 2019 and Mavaddat et al 2023^1,2^.

1. Variables

Cancer worry was assessed at baseline and after the risk disclosure by the Cancer Worry Scale (CWS) that consists of 6 items designed to measure the degree of worry or concern individuals experience related to the possibility of developing cancer. Participants rate their feelings on a scale, providing insights into the psychological impact of cancer risk assessment^3^.

Numeracy was evaluated by the validated Lipkus Numeracy Scale designed to measure an individual's numeracy skills, specifically their ability to comprehend and work with numerical information related to health. The scale includes items that assess basic numeracy, such as understanding percentages, probabilities, and quantitative health information^4^.

Personality traits were assessed using the Mini-IPIP brief personality assessment tool which consists of 20 items and is designed to measure the Big Five personality traits: Openness, Conscientiousness, Extraversion, Agreeableness, and Neuroticism. Participants responded to each item on a Likert-type scale, indicating the extent to which they agree or disagree with statements reflecting different aspects of personality^5,6^.

The specific psychological impact of personalized breast cancer risk estimation was evaluated using the 25-item Spanish version of the Multidimensional Impact of Cancer Risk Assessment Questionnaire (MICRA). This instrument provides a total score within the range of 0 to 105 and consists of three subscales: distress (0–30), uncertainty (0–45), and positive experiences (0–20). While initially designed to gauge the psychological impact of cancer genetic testing, we adapted the questionnaire for the purposes of this study, aiming to assess the psychological impact of personalized breast cancer risk estimation^7^.

The Decisional Conflict Scale includes 16 items and assesses an individual's uncertainty and difficulty in making decisions regarding health-related choices. It measures factors such as feeling uninformed, unclear values, and unsupported decision-making, providing insights into decisional conflict. In this instance, our focus was directed towards the decision-making process associated with participation in the PRiSma study^8^.

Anxiety after the disclosure was assessed by the State-Trait Anxiety Inventory which comprises two subscales, each with 20 items. The State Anxiety scale measures current anxiety levels, while the Trait Anxiety scale assesses a person's general or long-standing anxiety tendencies. This scale helps gauge anxiety levels before and after receiving risk information^9^.

Several custom-designed questions with Likert scale responses were employed to evaluate different aspects, including risk perception, satisfaction with disclosure, amount of information, utility of information, perceived benefit, recommendation to others, and understanding of multifactorial risk.

**References**

1. Mavaddat N, Ficorella L, Carver T, et al. Incorporating Alternative Polygenic Risk Scores into the BOADICEA Breast Cancer Risk Prediction Model. *Cancer Epidemiology, Biomarkers & Prevention*. 2023;32(3):422-427. doi:10.1158/1055-9965.epi-22-0756

2. Mavaddat N, Michailidou K, Dennis J, et al. Polygenic Risk Scores for Prediction of Breast Cancer and Breast Cancer Subtypes. *Am J Hum Genet*. 2019;104(1):21-34. doi:10.1016/j.ajhg.2018.11.002

3. Cabrera E, Zabalegui A, Blanco I. Versión española de la Cancer Worry Scale (Escala de Preocupación por el Cáncer: adaptación cultural y anlisis de la validez y la fiabilidad). *Med Clin (Barc)*. 2011;136(1):8-12. doi:10.1016/j.medcli.2010.04.015

4. Lipkus IM, Samsa G, Rimer BK. General performance on a numeracy scale among highly educated samples. *Med Decis Making*. 2001;21(1):37-44. doi:10.1177/0272989X0102100105

5. Baldasaro RE, Shanahan MJ, Bauer DJ. Psychometric properties of the mini-IPIP in a large, nationally representative  sample of young adults. *J Pers Assess*. 2013;95(1):74-84. doi:10.1080/00223891.2012.700466

6. Donnellan MB, Oswald FL, Baird BM, Lucas RE. The mini-IPIP scales: tiny-yet-effective measures of the Big Five factors of  personality. *Psychol Assess*. 2006;18(2):192-203. doi:10.1037/1040-3590.18.2.192

7. Cella D, Hughes C, Peterman A, et al. A brief assessment of concerns associated with genetic testing for cancer: The Multidimensional Impact of Cancer Risk Assessment (MICRA) questionnaire. *Health Psychology*. 2002;21(6):564-572. doi:10.1037//0278-6133.21.6.564

8. Urrutia M, Campos S, O’Connor A. [Validation of a Spanish version of the Decisional Conflict scale]. *Rev Med Chil*. 2008;136(11):1439-1447. doi:10.4067/s0034-98872008001100010

9. Buela-Casal G, Guillén-Riquelme A. Short form of the Spanish adaptation of the State-Trait Anxiety Inventory. *International Journal of Clinical and Health Psychology*. 2017;17(3):261-268. doi:10.1016/j.ijchp.2017.07.003
