# Supplementary material for: A randomized study of 2 risk assessment models for individualized breast cancer risk estimation
Source: J Natl Cancer Inst. 2025 Apr 1;117(8):1593–604. doi: 10.1093/jnci/djaf067 (PMC12342814; doi:10.1093/jnci/djaf067)
Supplement: djaf067_Supplementary_Data [file djaf067_supplementary_data.zip › djaf067_Supplementary_Data/Supplementary Tables.docx]

**Supplementary Table 2.** Changes in 10-year risk categories following the inclusion of breast density (BD), or BD and polygenic risk score (PRS) to the baseline 10y-BC risk estimation model.

|  | **With the addition of BD**    **n (%)** | **With the addition of BD and PRS**    **n (%)** |
| --- | --- | --- |
| **Change in 10-year risk-group** | **147 (24.7)** | **198 (33.4)** |
| **Increased risk-group** | **59 (9.9)** | **83 (14)** |
| Average to moderate | 43 (7.2) | 51 (8.6) |
| Average to high | 0 (0) | 2 (0.3) |
| Moderate to high | 16 (2.7) | 30 (5.1) |
| **Decreased 10-year risk-group** | **89 (14.8)** | **115 (19.4)** |
| Moderate to average | 85 (14.3) | 110 (18.6) |
| High to moderate | 3 (0.5) | 5 (0.8) |
| High to average | 0 (0) | 0 (0) |
| **No change in 10-year risk-group** | **446 (75.3)** | **395 (66.6)** |
| **Total** | **593** | **593** |

|  | **With the addition of BD**  **n (%)** | **With the addition of BD and PRS**    **n (%)** |
| --- | --- | --- |
| **Change in lifetime risk-group** | **141 (23.8)** | **205 (34.5)** |
| **Increased risk-group** | **84 (14.2)** | **124 (20.9)** |
| Average to moderate | 69 (11.6) | 88 (14.8) |
| Average to high | 0 (0) | 7 (1.2) |
| Moderate to high | 15 (2.5) | 29 (4.9) |
| **Decreased lifetime risk-group** | **57 (9.6)** | **81 (13.6)** |
| Moderate to average | 57 (9.6) | 78 (13.1) |
| High to moderate | 0 (0) | 3 (0.5) |
| High to average | 0 (0) | 0 (0) |
| **No change in lifetime risk-group** | **452 (76.2)** | **388 (65.5)** |
| **Total** | **593** | **593** |

**Supplementary Table 3.** Changes in lifetime risk categories following the inclusion of breast density (BD), or BD and polygenic risk score (PRS) to the baseline lifetime risk estimation model.

**Supplementary Table 4.** Predictors of higher MICRA score associated to breast cancer risk assessment.

| **Multivariable generalized linear model** | **Estimate** | **SE** | **p-value** |
| --- | --- | --- | --- |
| Delivery model  (Pre-recorded video vs In-Person) | 0.32 | 0.09 | **<0.001** |
| Breast cancer risk group  (moderate vs average) | -0.24 | 0.09 | **0.01** |
| Age (1-year increment) | -0.01 | 0.01 | 0.98 |
| Conscientiousness (high vs low) | -0.02 | 0.02 | 0.21 |
| Extraversion (high vs low) | 0.03 | 0.02 | 0.07 |
| Neuroticism (high vs low) | 0.02 | 0.02 | 0.26 |
| Imagination (high vs low) | -0.01 | 0.02 | 0.48 |
| Agreeableness (high vs low) | -0.03 | 0.02 | 0.09 |
| Numeracy skills# (high vs low) | -0.06 | 0.02 | **0.01** |

*SE: Standard Error; #Measured with the LIPKUS scale
